# Pulmonary Hypertension in Patients With Heart Failure With Mid-Range Ejection Fraction

**DOI:** 10.3389/fcvm.2021.694240

**Published:** 2021-07-09

**Authors:** Micha T. Maeder, Lukas Weber, Marc Buser, Roman Brenner, Lucas Joerg, Hans Rickli

**Affiliations:** Cardiology Department, Kantonsspital St. Gallen, St. Gallen, Switzerland

**Keywords:** pulmonary hypertension, heart failure, post-capillary, left ventricular ejection fraction, mid-range, mildly reduced, right heart catheterization

## Abstract

Pulmonary hypertension (PH) is common in patients with heart failure (HF). The role of PH in patients with HF with reduced (HFrEF) and preserved (HFpEF) left ventricular ejection fraction (LVEF) has been extensively characterized during the last years. In contrast, the pathophysiology of HF with mid-range LVEF (HFmrEF), and in particular the role of PH in this context, are largely unknown. There is a paucity of data in this field, and the prevalence of PH, the underlying mechanisms, and the optimal therapy are not well-defined. Although often studied together there is increasing evidence that despite similarities with both HFrEF and HFpEF, HFmrEF also differs from both entities. The present review provides a summary of the current concepts of the mechanisms and clinical impact of PH in patients with HFmrEF, a proposal for the non-invasive and invasive diagnostic approach required to define the pathophysiology of PH and its management, and a discussion of future directions based on insights from mechanistic studies and randomized trials. We also provide an outlook regarding gaps in evidence, future clinical challenges, and research opportunities.

## Introduction

Pulmonary hypertension (PH) in patients with left heart diseases is the most common form of PH (1). The presence of PH in this context typically reflects an advanced disease stage with exhausted compensatory mechanisms, which is associated with exercise intolerance and a poor prognosis (2). Thus, PH is a manifestation of heart failure (HF). In patients with HF with reduced left ventricular ejection fraction (LVEF; HFrEF) PH is a common feature in the decompensated state that is often reversible following appropriate therapy. In patients with advanced HFrEF, PH may become chronic and thereby is a marker of poor prognosis (3, 4). Intense research during the last decade has revealed that PH may be even more common in patients with HF with preserved LVEF (HFpEF) (2). There is increasing evidence from recent studies using invasive hemodynamics with or without exercise in combination with detailed echocardiographic assessments that the pathophysiology underlying PH in HFpEF is complex and differs from that in HFrEF (5). In contrast, our understanding of the relatively new disease entity of HF with mid-range LVEF (or “mildy reduced” LVEF; HFmrEF) is still evolving, and the pathophysiology and clinical impact of PH in this context have not been defined yet (6). In this review, we discuss the potential role of PH in HFmrEF, highlight the diagnostic challenges, propose a clinical approach, and briefly summarize the therapeutic options in these patients with an outlook to potential future developments. We have to acknowledge that there is still a paucity of data on PH in HFmrEF. Before HFmrEF was clearly defined as a distinct entity, these patients were often included in HFrEF or HFpEF studies on pathophysiology and therapy. We therefore also discuss HFmrEF in the context of concepts regarding PH in HFrEF vs. HFpEF.

## Definition of HFmrEF

The relatively new entity of HFmrEF has been introduced by the 2016 European Society of Cardiology (ESC) guidelines on the diagnosis and management of HF (7). In these guidelines, HFmrEF includes the LVEF range between 40 and 49% while patients with LVEF <40% by definition have HFrEF and those with LVEF ≥50% have HFpEF (Table 1). A slightly different definition of HFrEF and HFmrEF has been proposed in the recently published Universal Definition and Classification of Heart Failure (10): HFrEF: LVEF ≤ 40% rather than <40%, HFmrEF: LVEF 41–49% rather than 40–49%. The definition of HFpEF remains unchanged: LVEF ≥50%. In this review, we adopt this new definition. However, when discussing studies specifically looking at HFmrEF, we must be aware that often the “old” LVEF range of 40–49% was applied. The rationale underlying the creation of the HFmrEF category had been as follows: on the one hand, the established HFrEF pharmacotherapy is based on studies that included patients up to an LVEF of 40% (not up to 50%), and on the other hand, it has been realized that in the large randomized “HFpEF studies,” which included patients with LVEF ≥40%/45% (rather than only ≥50%), those with LVEF <50% responded differently to several pharmacological interventions when compared to those with LVEF ≥50% (7). The 2016 ESC guidelines state that apart from the LVEF criteria the same additional criteria are required for the diagnosis of both HFpEF and HFmrEF (Table 1) (7). More recently, a new algorithm for the diagnosis of HFpEF was proposed in an ESC position paper (HFA-PEFF score) (8), and a somewhat different diagnostic score (H2FPEF score; gold standard: invasive exercise hemodynamics) was published around the same time by the HFpEF experts from the Mayo clinic (9). Whether or not these approaches for the diagnosis of HFpEF can also be applied to make a diagnosis of HFmrEF, has not been explicitly addressed. In 2021, new ESC guidelines on HF are expected, and some of these aspects may be described more clearly.

**Table 1:** Definition of heart failure (HF) with mid-range (HFmrEF) vs. HF with reduced (HFrEF) and HF with preserved (HFpEF) left ventricular ejection fraction.

	**HFrEF**	**HFmrEF**	**HFpEF**
LVEF^a^	≤ 40%	41–49%	≥50%
Definition ESC guidelines 2016 (7)	Symptoms ± signs	1. Symptoms ± signs	
		2. NT-proBNP >125 ng/l or BNP >35 ng/l	
		3. LV hypertrophy/LA dilation or significant LV diastolic dysfunction: LVMI ≥115 g/m^2^ (males) or 95 g/m^2^ (females), LAVI 34 ≥ml/m^2^, E/e′≥13, e′ (average from septal and lateral annulus) <9 cm/s	
Definition ESC position paper 2019 (8)		Not explicitly included	HFA-PEFF score:^b^ ≥5 points: HFpEF ≤ 1 points: HFpEF unlikely 2–4 points: functional test: non-invasive diastolic stress test or invasive stress test (Gold standard: mPAWP ≥15 mmHg at rest or/and ≥25 mmHg on exercise)
Definition Mayo 2018 (9)		Not included	H2FPEF score:^c^ doubling of the probability of HFpEF with each one-point increase

a *LVEF cut-offs adopted from the 2021 Universal Definition and Classification of Heart Failure (10)*.

b *Heart Failure Association (HFA)-PEFF: score: composed of (a) septal or lateral peak early diastolic mitral annular velocity by tissue Doppler (e′), ratio of peak early diastolic transmitral velocity by pulsed wave Doppler (E) to e′ (E/e′), peak tricuspid regurgitant velocity, estimated systolic pulmonary artery pressure (sPAP), or global longitudinal strain, (b) left atrial volume index (LAVI), left ventricular (LV), mass index (LVMI), or relative wall thickness, and (c) B-type natriuretic peptide (BNP), or N-terminal proBNP (NT-proBNP). Cut-offs depend on age (<75 vs. ≥75 years) and cardiac rhythm (sinus rhythm vs. atrial fibrillation). Values between 0 and 6*.

c *H_2_FPEF score: composed of: Heavy: body mass index >30 kg/m^2^ (two points), hypertensive: two or more antihypertensive drugs (1 point), atrial fibrillation: paroxysmal or persistent (three points), pulmonary hypertension (sPAP) >35 mmHg (1 point), elder: age >60 years (1 point), and filling pressure: E/e′ >9 (1 point). values between 0 and 9. mPAWP, mean pulmonary artery wedge pressure*.

Notably, in both the new ESC HFA-PEFF score (8) and the Mayo clinic H2FPEF score (9) a measure of PH is an item contributing to the diagnosis of HFpEF. This highlights that PH is a common feature in HFpEF. However, the non-critical use of this criterion may be misleading in certain situations. The rationale to use PH as a marker of HFpEF is based on the fact, that this typically is a reflection of post-capillary PH in the context of advanced left ventricular diastolic and left atrial (LA) dysfunction. However, sometimes this assumption may not be correct, and a preserved or mid-range LVEF may co-exist with a form of PH that is unrelated to a left heart pathology.

## Hemodynamic definition of pulmonary hypertension in HF

In patients with left heart disease, PH is most often a reflection of elevated LA pressure and pulmonary artery wedge pressure, respectively, i.e., post-capillary PH (group 2 PH) (1, 2). According to the 2015 ESC/European Respiratory Society (ERS) guidelines, any PH is defined as a mean pulmonary artery pressure (mPAP) ≥25 mmHg. Post-capillary PH is defined by a mean pulmonary artery wedge pressure (mPAWP) >15 mmHg (pre-capillary PH: mPAWP ≤ 15 mmHg) (11). If PH is driven by mPAWP elevation alone (no relevant pulmonary vascular disease), this is referred to as isolated post-capillary PH (IpcPH), which is defined as mPAP ≥25 mmHg, mPAWP>15 mmHg, pulmonary vascular resistance (PVR) ≤ 3 Wood units (WU), and/or diastolic pressure gradient (DPG) <7 mmHg. If there is an associated pulmonary vascular component of PH (typically as a reaction of the pulmonary vasculature to a longstanding and substantial mPAWP elevation), this is referred to as combined pre- and post-capillary PH (CpcPH), which is defined as mPAP ≥25 mmHg, mPAWP>15 mmHg, and PVR >3 WU and/or DPG ≥7 mmHg (11). It has been recognized that DPG values are often low and even negative and discordant to PVR, which leads to many unclassifiable patients when applying the original 2015 ESC/ERS criteria. In addition, in contrast to PVR data on the prognostic value of the DPG have been inconsistent. Therefore, the PVR criterion is preferred (12).

The 2018 PH World Symposium has proposed a new PH definition, which aims to overcome the above-mentioned limitations of the 2015 definition and to consider new data on the normal range of pulmonary pressures. This new definition is under intense discussion, however, and there are no new PH guidelines yet. According to this approach, pre-capillary PH is defined as mPAP >20 mmHg (new cut-off), mPAWP ≤ 15 mmHg, and PVR ≥3 WU (new compulsory criterion) (13). Post-capillary PH is defined as mPAP >20 mmHg (new cut-off) and mPAWP >15 mmHg (IpcPH: PVR <3 WU, CpcPH: PVR ≥3 WU; i.e., the PVR criterion has been slightly modified, and the DPG criterion has been dropped for the above-mentioned reason) (14). The rationale for this new PH definition is as follows: (1) studies have shown that the upper limit of a normal mPAP is approximately 20 mmHg, and mortality is already increased in patients with mPAP >20 mmHg. (2) The introduction of the PVR ≥3 WU criterion for the definition of pre-capillary PH makes sure that there is really pulmonary vascular disease rather than increased flow. (3) A single criterion (i.e., PVR ≥3 WU, no DPG criterion) for the differentiation between IpcPH and CpcPH ensures an unequivocal definition in case of discordant PVR and DPG criteria (13, 14).

## Prevalence of PH in HFpEF and HFmrEF

In the largest study from a catheterization laboratory database (*n* = 10,023), 46% of all patients undergoing right heart catheterization had post-capillary PH (74% of all patients with PH) (15), and 39% of them had HFrEF, 56% had HFpEF, and in 5% the LVEF was not recorded. In this study, the LVEF cut-off for the differentiation between HFrEF and HFpEF was 45%, i.e., the HFmrEF group was not separated (15). Although there is a bimodal distribution of LVEF in HF (16), it is likely that there was a sizeable group of patients with PH in the context of HFmrEF. Cohort studies looking at unselected HF patients, i.e., patients with HF but not necessarily PH, revealed an HFmrEF prevalence of 13–24% (17–20). Mortality of HFmrEF patients was intermediate between HFrEF and HFpEF in some (20) and similar to HFrEF but better than in HFpEF in other studies (17). The proportion of HFmrEF patients among group 2 PH patients is unknown, and the prevalence of PH among unselected patients with HFmrEF and the prognosis of patients with HFmrEF and PH not known either. The estimation of the prevalence of PH in HF is difficult because a reliable diagnosis of PH by echocardiography is not possible in cross-section studies, and all invasive studies suffer from a very substantial referral bias since the indication for right heart catheterization in these patients most likely was based on evidence of PH in the echocardiogram.

In one study using a non-invasive PH definition [systolic pulmonary artery pressure (sPAP) >35 mmHg, i.e., peak tricuspid regurgitant velocity (TRV) ≈2.9 m/s assuming a normal central venous pressure], a high PH prevalence of 83% was found among 244 HFpEF (LVEF ≥50%) patients from a community based study (21). Many of the large HFpEF intervention studies also included patients who now meet the definition of HFmrEF. In the Prospective Comparison of ARNI With ARB Global Outcomes in HF With Preserved Ejection Fraction (PARAGON-HF) trial, the LVEF cut-off for inclusion was ≥45%. Study inclusion was based on the LVEF derived from a screening echocardiogram (LVEF reported by the study site). In the echo substudy of the trial (*n* = 1,079), the median LVEF according to secondary core lab analysis was 59%, and LVEF was ≥50% in 79%, 40–50% in 18%, and <40% in 3% of patients. The prevalence of PH (defined as peak TRV >2.9 m/s) in this PARAGON-HF subgroup was 31% (22). The mean estimated sPAP was 34 mmHg (peak TRV 2.7 m/s, plus a value for the estimated central venous pressure) (22). This was similar to the echo substudies of the Irbesartan in Heart Failure With Preserved Ejection Fraction (I-PRESERVE) (≈37 mmHg) (23) and Treatment of Preserved Cardiac Function Heart Failure With an Aldosterone Antagonist (TOPCAT) (≈38 mmHg) studies (24) where the same LVEF cut-off of ≥45% was used as inclusion criterion. It is obvious that all these large “HFpEF trials” included a certain proportion of HFmrEF patients but peak TRV values were not reported separately for patients with LVEF ≥50 vs. 45–49%. Such data were shown however in an analysis of the Trial of Intensified Medical therapy in Elderly patients with Congestive Heart Failure (TIME-CHF), where the mean peak TRV in HFrEF (*n* = 289), HFmrEF (*n* = 82), and HFpEF (*n* = 85) were ≈2.9, ≈2.9, and ≈3.0 m/s, respectively (no difference) (25). Thus, assuming a normal distribution of peak TRV values 50% of patients in all LVEF strata formally had an at least intermediate probability of PH in TIME-CHF and may have had some degree of PH. This is probably an overestimation as a peak TRV of 2.9 m/s without indirect signs of PH represents the lower margin of the intermediate probability stratum. Still, the data suggest that PH is equally common in HFmrEF as in HFpEF and HFrEF. On the other hand, it must be realized, however, that the TIME-CHF population was a highly selected one. All patients had been hospitalized before inclusion, and high N-terminal-pro-B-type natriuretic peptide (NT-proBNP) values (>400 ng/l for patients with age 60–74 years, >800 ng/l for those age >75 years) were required for study inclusion (26) [cf. PARAGON study: >200 ng/l for patients in sinus rhythm, and >600 ng/l for those in atrial fibrillation (27)]. Given that pulmonary pressures are related to natriuretic peptides (28) this inclusion criterion may have led to a selection of patients with high likelihood of PH. Accordingly, up to 30–50% of patients with HFmrEF may have some form of PH.

## Clinical, echocardiographic, and biochemical characteristics of patients with HFmrEF

The primary driver of PH in any type of left heart disease is an elevation in LA pressure, which in turn depends on the properties of the left ventricle, the function of the mitral valve, and the compliance of the left atrium. Data examining this pathopathopysiology specifically in HFmrEF are sparse. To understand the mechanism of PH in HFrEF, we first discuss available studies looking at the clinical characteristics and echocardiographic features in HFmrEF, and then look at the mechanistic studies on PH in HFrEF and HFpEF as a basis to speculate about the situation in HFmrEF.

In terms of clinical characteristics, HFmrEF patients more closely resemble the HFrEF rather than the HFpEF group (younger age, less females, more ischemic heart disease, less atrial fibrillation) (20). The available data on cardiac structure and function in HFmrEF suggest that these patients exhibit a phenotype, which is overall intermediate between HFrEF and HFpEF (25, 29–31). However, such data is limited, and it is actually unknown which different cardiac pathologies associated with a mid-range LVEF the patients really had who were included in the larger cross-sectional studies. In addition, the LVEF range of 41–49% is relatively narrow, and the HFmrEF group includes patients with stable LVEF but also patients with HFrEF and improved LVEF and patients with HFpEF and worsened LVEF (32). Notably, this trajectory of LVEF is important in terms of prognosis (18, 19, 32); in particular, the change from HFmrEF to HFrEF is a marker of an adverse outcome (19). In this context, the presence of coronary artery disease has been found to be an important mechanism related to a reduction in LVEF and change in LVEF category (33).

In the well-characterized TIME-CHF population, the ischemic HF etiology was similarly common in HFmrEF as in HFrEF, and the atrial fibrillation (AF) prevalence was similar in HFmrEF and HFpEF (25). Left ventricular dimension, mass and geometry in HFmrEF patients were intermediate between HFrEF and HFpEF. Despite differences in LVEF by definition, right ventricular (RV) function, and the peak TRV were similar in all three LVEF categories (25). In a study by Ghio et al. (34) the left ventricular end-diastolic volume index and the prevalence of significant mitral regurgitation (MR) in HFmrEF were similar as in patients with HFrEF and thereby larger/higher than in HFpEF. In contrast, right ventricular function expressed as tricuspid annular plane systolic excursion (TAPSE) was somewhat lower in HFmrEF and HFpEF compared to HFrEF.

The biomarker profile in HFmrEF is also characterized by intermediate plasma concentrations of natriuretic peptides and a pattern of biomarkers that includes features of both HFpEF and HFrEF, i.e., markers of both inflammation and cardiac stretch, whereas in HFpEF, biomarkers were related to inflammation, and in HFrEF, biomarkers were related to cardiac stretch (35).

## Pathophysiology of PH in HFmrEF

There is evidence for substantial differences in the pathophysiology of PH between patients with HFpEF and HFrEF (5). In HFpEF, concentric remodeling/hypertrophy and increased diastolic stiffness represent the hallmarks of the pathophysiology. Many HFpEF patients have diabetes, obesity, and hypertension, and it has been suggested that these comorbidities activate pro-inflammatory pathways leading to increased collagen deposition (36). In contrast, HFrEF patients are characterized by eccentric remodeling/hypertrophy and high wall stress. Patients with HFmrEF have intermediate left ventricular volumes, mass, and relative wall thickness, and values for the peak early mitral annular velocity (e′) (25). Left atrial dysfunction is the key mechanism contributing to LA pressure and mPAWP and mPAP elevation. Left atrial remodeling differs between patients with HFpEF and HFrEF with less atrial dilation but higher atrial stiffness in HFpEF (37). Left atrial volume index is highest in patients with HFrEF, lowest in those with HFpEF, and intermediate in HFmrEF (30), suggesting an intermediate type of remodeling. Patients with HF irrespective of LVEF exhibit a significantly reduced LA strain at rest and during exercise when compared to patients with dyspnea of non-cardiac origin (30, 38). In a HF population with a broad LVEF spectrum there was overall an inverse correlation between higher LA volume index and lower LA strain, which was relatively moderate however. There was also correlation between lower LA strain during exercise and lower peak exercise cardiac output and peak oxygen consumption (30). The HFmrEF patients had the highest LA strain at rest when compared to HFpEF and HFmrEF but a blunted response to exercise with exercise with LA strain values being intermediate between HFpEF and HFrEF (30).

There are two factors with an important interaction with LA function and thereby promoting PH: MR and AF. In HFrEF, various degrees of functional MR are common and predict prognosis (39). In these patients, MR results from an imbalance between tethering and closing forces in the context of the dilatation and distorted geometry the left ventricle (40). In contrast, HFpEF patients are characterized by “atrial” functional MR, i.e., MR due to annulus dilatation in the context of LA dilation, typically in the context of AF (41). Mitral regurgitation can be dynamic in both HFrEF and HFpEF as shown in exercise studies (31, 41). In HFmrEF both forms of functional MR likely play role, depending on the predominant type of LV remodeling. In a recent study, significant MR at rest was found in 15% of patients with HFpEF, in 27% of those with HFmrEF, and in 47% of those with HFrEF. Importantly, exercise elicited worsening of MR in all HF categories with at least moderate MR in 35, 41, and 60% of HFpEF, HFmrEF, and HFrEF patients during exercise (31). In any type of HF, there is vicious cycle between MR and LA remodeling. The same applies for AF and LA remodeling and MR, respectively. In HFmrEF, AF is similarly common as in HFpEF and more prevalent than in HFrEF (20). Importantly, presence of AF (either by AF per se and/or mediated by the AF-associated structural changes) has substantial impact on hemodynamics, in particular on the relationship between LVEDP and mPAWP (42–44). The AF burden (paroxysmal vs. permanent) is a marker of the hemodynamic derangement in HFpEF (45), and the same may apply for HFmrEF. In sinus rhythm, LVEDP is typically similar or somewhat higher than mPAWP because MR is typically mild, LA function is only moderately reduced, and there is an effective atrial contraction. In contrast, in AF substantial LA dysfunction, higher degrees of MR and absence of LA contraction lead to high V waves and higher mPAWP than LVEDP. Patients with AF typically have worse hemodynamics and those in sinus rhythm with higher mPAWP, mPAP, and PVR and higher prevalence of PH and CpcPH (44).

Apart from differences in the mechanisms of LA pressure and mPAWP elevation, there is evidence for important LVEF-dependent differences in the pathobiology of the pulmonary vasculature in response to a certain LA pressure and mPAWP, respectively (46). In a cross-sectional study, patients with HFpEF have been shown to have a higher PVR for a given mPAWP, i.e., a higher likelihood of CpcPH, than patients with HFrEF (46). The anatomical substrate for the pre-capillary component of PH in CpcPH in HF is still not well-defined. It has been assumed that there are similar vascular changes as in pulmonary arterial hypertension. However, a recent post-mortem analysis of lung specimens from patients with HFrEF (*n* = 55) and HFpEF (*n* = 53) with PH (all with documented sPAP ≥40 mmHg; 30 with right heart catheterization data: mPAP = 40 mmHg, mPAWP = 25 mmHg, PVR = 3.9 WU) has revealed global (arteries, veins, indeterminate vessels) pulmonary vascular remodeling (47). There was substantial intimal thickening and medial hypertrophy of pulmonary veins (“pulmonary vein arterialization”) resembling the changes seen in pulmonary veno-occlusive disease, and the extent of medial hypertrophy in the pulmonary arteries was related to the extent of venous intimal thickening but not arterial thickening suggesting that arterial medial hypertrophy may develop secondary to venous remodeling. The medial thickness of arteries and the intimal thickness of arteries and veins tended to be more severe in HFpEF vs. HFrEF, and intimal thickness of veins was significantly more severe in HFpEF compared to HFrEF. The severity of PH expressed as transpulmonary gradient and PVR was correlated most strongly with venous and small indeterminate vessel intimal thickening as was the impairment in diffusion capacity of the lung (47). In that study, HFrEF was defined as LVEF <50%, and HFpEF as LVEF ≥50%. Thus, HFmrEF was included in the HFrEF group. The 75^th^ percentile for LVEF in HFrEF was 35%, and thus some patients with HFmrEF may have been included. The overall similar pattern of pulmonary vascular remodeling in HFpEF and HFrEF suggest that these observations likely also apply for HFmrEF. The underlying pathophysiology in humans is not clearly defined but endothelial injury due to barotrauma (alveolar-capillary stress failure) and subsequent remodeling under the influence of several mediators seems to be of paramount importance (5). There is evidence from a rat HFpEF model that the metabolic syndrome may promote the development of pulmonary vascular disease in HFpEF (48). Given the similar prevalence of Diabetes in HFpEF and HFmrEF (20, 25) this may be relevant to the pathophysiology of pulmonary vascular disease also in HFmrEF.

Right ventricular dysfunction is a strong predictor of prognosis in HFrEF (49) and HFpEF (49). The RV is very sensitive to pressure overload. Therefore, adaption of the RV to PH is crucial (50). This seems to be particularly relevant for HFpEF and HFmrEF, while in HFrEF intrinsic RV dysfunction also plays an important role. In important study by Ghio (34), ischemic HF etiology, non-sinus rhythm, and high heart rate were related to TAPSE in HFrEF, while in HFpEF pulmonary pressure was the strongest predictor of TAPSE, and the same was true for patients with HFmrEF. In this context, the concept of RV to pulmonary artery (PA) coupling is of critical importance, i.e., ability of the RV to cope with the increased afterload. Classically, RV to PA coupling is described by RV pressure volume analysis, which is a cumbersome technique that is rarely applied in clinical practice. The RV to PA coupling is defined as the ratio between RV end-systolic elastance (Ees; end-systolic RV pressure divided by end-systolic volume) and arterial elastance (Ea; RV end-systolic pressure divided by stroke volume). Normally, Ees/Ea [which can also be expressed as RV ejection fraction/(1-RV ejection fraction)], is around 1.5 and can be reduced to approximately 0.8 before RV dilatation occurs (“uncoupling”) (50). For clinical practice, the ratio of TAPSE to estimated sPAP (TAPSE/sPAP) has been proposed as non-invasive surrogate for Ees/Ea (51). In a large HFpEF population undergoing detailed non-invasive and invasive hemodynamic evaluation, those in the lowest TAPSE/sPAP tertile had the worst hemodynamics including the worst RV function, the highest right atrial pressure, mPAP, and PVR, and the highest proportion of CpcPH (52). Similarly, another study found lower TAPSE/sPAP ratio in CpcPH vs. IpcPH in both patients with HFrEF and HFpEF (53). This study used an LVEF cut-off of 45% to differentiate between HFrEF and HFpEF (53). Thus, patients with HFmrEF were included but separate data are not available. However, in an exercise echocardiography study LA dynamics expressed as changes in LA strain during exercise were correlated to TAPSE/sPAP not only in HFrEF and HFpEF but also in HFmrEF (30) suggestig that TAPSE/sPAP may be marker of RV dysfunction and high mPAP, mPAP, and PVR due to LA myopathy and functional MR with high pulsatile load also in HFmrEF.

## Phenotypes of HFmrEF with PH

**Figure 1:**
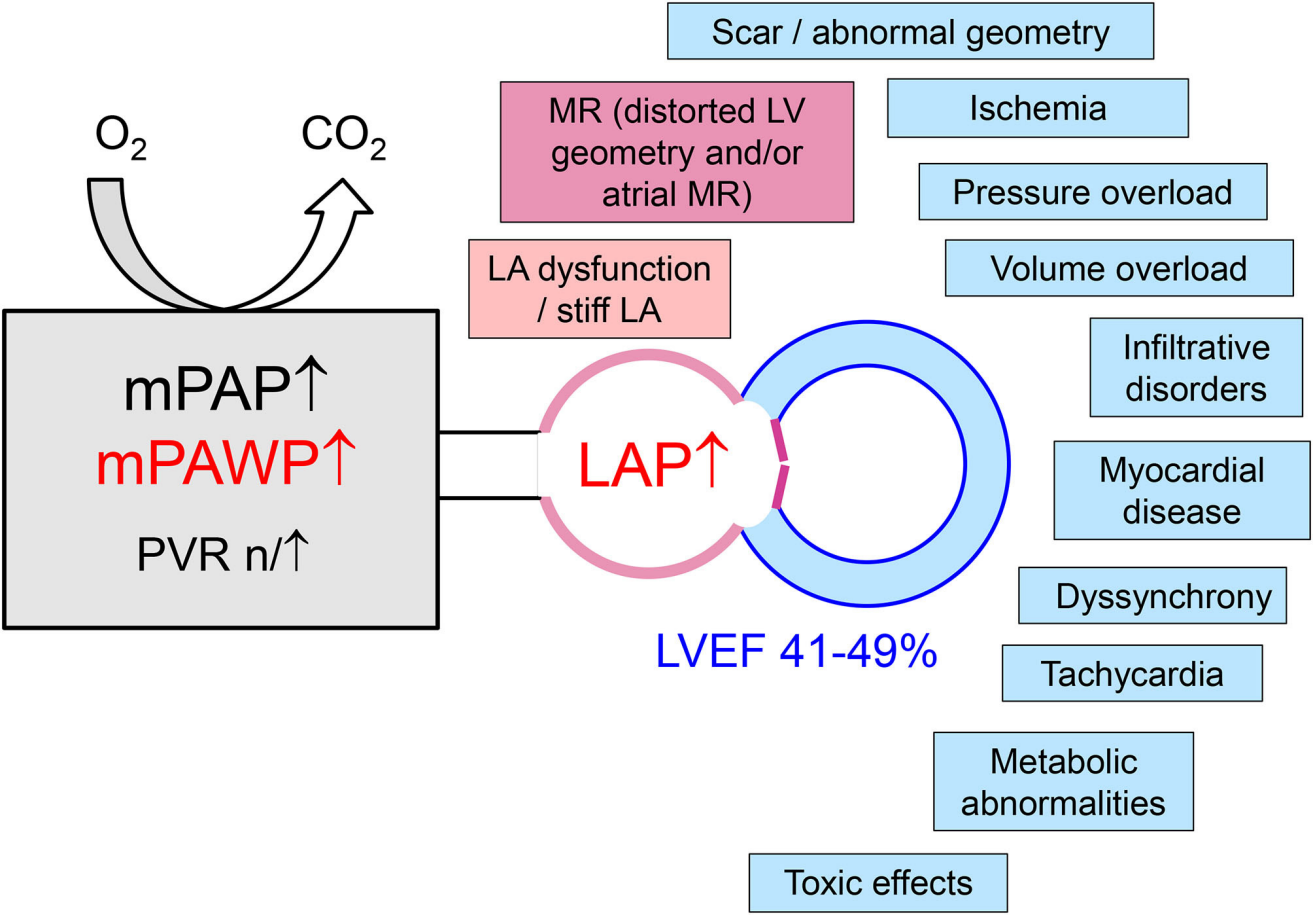
Mechanisms of left atrial pressure (LAP) elevation in patients with pulmonary hypertension in the context of left heart disease with a left ventricular ejection (LVEF) in the mid-range of 41–49%. LA, left atrial/atrium; LV, left ventricular; mPAP, mean pulmonary artery pressure; mPAWP, mean pulmonary artery wedge pressure; MR, mitral regurgitation; PVR, pulmonary vascular resistance.

The HFmrEF group is a difficult one since the LVEF spectrum is very narrow (41–49%), and assessment of LVEF in clinical practice is associated with substantial variability (6). There is a large number of disease entities potentially presenting with a HFmrEF phenotype and also PH. Principally, most of the specific HFpEF etiologies listed in the most recent ESC position paper on HFpEF (8) can also result in HFpEF. Coronary artery disease with a previous moderate myocardial infarction is a relatively common etiology of HFmrEF, and the documented change from HFpEF to HFmrEF in the context of coronary artery disease is a marker of an unfavorable prognosis (33). Apart from coronary artery disease, a large number of non-ischemic etiologies may play a role including infiltrative diseases and hypertrophic cardiomyopathies. In this context, a frank reduction in LVEF (as opposed to “only” reduced tissue Doppler/strain) represents an advanced disease stage. A more detailed discussion of these specific entities is beyond the scope of the present review however. Although cohort studies suggest that overall HFmrEF patients are characterized by a structural and pathophysiological phenotype, which is intermediate between HFrEF and HFpEF (25, 29), the existence of a number of different phenotypes is very likely but this has not been analyzed in detail so far. Still, the importance of the different mechanism contributing to PH as discussed in the previous section may vary. A schematic representation of different entities/mechanisms leading to LA pressure elevation in PH in HFmrEF is shown in Figure 1. An incomplete list of some proposed distinct and important entities summarized under the HFmrEF umbrella and the possible mechanisms of PH is presented in Table 2. In contrast to previous more restrictive diagnostic criteria for HFpEF (54), the most recent ESC consensus explicitly states that the HFpEF spectrum not only includes the classical “lone” HFpEF form but also specific etiologies (e.g., cardiomyopathies) and patients with primary valve disease as long as the definition criteria are met (Table 1) (8). Patients with primary valve disease (i.e., typically severe aortic stenosis or severe organic MR) who have an LVEF between 41 and 49% and evidence of PH are in an advanced disease stage with relevant “cardiac damage” (Table 2). Evaluation and management of such patients will not be discussed in this review article but this can be found elsewhere (55, 56).

**Table 2:** Pulmonary hypertension (PH) in Heart Failure with mid-range left ventricular Ejection Fraction (HFmrEF): different disease entities and mechanisms (please also see text).

	**Disease characteristics**	**PH mechanism**	**Diagnostic approach**	**Treatment**
“Lone HFmrEF”	Classical form of HFmrEF in the context of obesity, hypertension and diabetes	LA pressure elevation due to systolic and diastolic LV dysfunction, functional (atrial) MR, LA dysfunction	• TTE including tissue Doppler/strain: anatomy, extent of LV systolic and diastolic dysfunction, LA dimensions, MR mechanism/severity • RHC	• Anticoagulation in atrial fibrillation • Loop diuretics, ARB, or ARNI (typically in women), MRA • Intraatrial shunt device for selected patients
Coronary artery disease	LV dysfunction after a previous infarct or due to chronic ischemia with hibernating myocardium, typically with functional MR	LA pressure elevation due to systolic and diastolic dysfunction, moderate/severe functional MR	• TTE, TOE: regional LV function, extent/mechanism of MR • Cardiac MRI: myocardial viability • coronary angiography: treatable ischemia • RHC	• Revascularization if possible • ARB (ARNI), MRA, betablocker, loop diuretic
Hypertrophic cardiomyopathy	LV hypertrophy and dysfunction with/without dynamic LVOT obstruction, functional MR, atrial fibrillation	LA pressure elevation due to systolic and diastolic LV dysfunction, functional MR, LA dysfunction	• TTE including tissue Doppler/strain: anatomy, extent of LV systolic and diastolic dysfunction, LVOT obstruction, MR • Coronary angiography: options for alcohol ablation • RHC	• Betablockers, verapamil, diltiazem • Surgical myectomy/alcohol ablation in presence of significant LVOT obstruction • Careful use of diuretics • If available: mavacamten (cardiac myosin inhibitor)
Specific cardiomyopathy, e.g., amyloidosis, sarcoidosis, scleroderma	LV infiltration and/or scarring with systolic and diastolic dysfunction, LA dysfunction, atrial fibrillation	LA pressure elevation due to systolic and diastolic LV dysfunction, LA dysfunction, functional (atrial) MR, secondary, and/or primary pulmonary vascular disease	• TTE including tissue Doppler/strain: anatomy, extent of LV systolic and diastolic dysfunction, MR • Search for specific etiologies using cardiac MRI, bone scintigraphy, positron emission tomography	• ARB (ARNI), MRA, betablocker, loop diuretics • Specific treatment of underlying disease (e.g., immunosuppression, tafamidis)
Tachycardia-mediated cardiomyopathy	LV dysfunction due to sustained tachycardia, LV and LA dilatation	LA pressure elevation due to systolic and diastolic dysfunction, functional (atrial) MR, LA dysfunction	• TTE including tissue Doppler/strain: anatomy, extent of LV systolic and diastolic dysfunction, LA dimensions, MR • Cardiac MRI: myocardial viability, evidence for specific disease • RHC • Coronary angiography in selected cases	• Anticoagulation • ARB (ARNI), MRI, Betablocker • Rhythm control (amiodarone, cardioversion, catheter ablation)
Valvular heart disease (after correction of valve stenosis/ regurgitation)	Persistent LV systolic and diastolic dysfunction late after correction of valve stenosis/regurgitation with/without pulmonary vascular disease	LA pressure elevation due to systolic and diastolic dysfunction, pulmonary vascular disease (elevated PVR)	• TTE including tissue Doppler/strain: anatomy, extent of LV systolic and diastolic dysfunction, LA dimensions • TOE for the exclusion of paravalvular leak etc. • RHC	• ARB (ARNI), MRA, betablocker, loop diuretic
Aortic stenosis	Advanced form of chronic severe aortic stenosis with reduced LVEF	LA pressure elevation due to systolic and diastolic dysfunction, MR, LA dysfunction, secondary pulmonary vascular disease	• TTE: severity of AS, LV systolic and diastolic dysfunction, LA size, MR • RHC • Coronary angiography	• Diuretics • ACE inhibitor • Aortic valve replacement if truly severe aortic stenosis
Mitral regurgitation	Advanced form of severe primary MR with reduced LVEF	LA pressure elevation due to systolic and diastolic dysfunction, and severe MR	• TTE and TOE: severity and mechanism of MR, LV dimensions, systolic and diastolic dysfunction, LA size • RHC • Coronary angiography	• Diuretics, ACE inhibitor, ARB • Mitral valve repair if severe primary MR

*ACE inhibitor, angiotensin converting enzyme inhibitor; ARB, angiotensin receptor blocker; ARNI, angiotensin receptor neprilysin inhibitor; LA, left atrial; LV, left ventricular; LVOT, left ventricular outflow tract; mPAP, mean pulmonary artery pressure; mPAWP, mean pulmonary artery wedge pressure; MR, mitral regurgitation; MRA, mineralocorticoid receptor blocker; MRI, magnetic resonance imaging; PVR, pulmonary vascular resistance; RHC, right heart catheterization; TOE, transoesophageal echocardiography; TTE, transthoracic echocardiography*.

## Diagnostic approach

Patients with a mildly reduced LVEF, i.e., between 41 and 49%, and evidence of possible PH represent a diagnostic challenge because a broad spectrum of disease mechanisms and hemodynamic patterns can be hidden behind this constellation (Figure 1, Table 2). In any case, the presence of PH is a marker of a serious problem, be it the consequence of the left heart disease or an independent entity (57), and therefore always requires a careful evaluation. The non-invasive diagnosis of PH by echocardiography remains difficult (1, 2). The peak TRV cannot always be measured in a reliable manner, and even if so, the correlation with the true sPAP is limited at least in certain settings (58). Guidelines recommend estimating the probability of PH using both peak TRV and indirect signs of PH (i.e., RV dilatation, flattening of the interventricular septum, short RV outflow tract acceleration time, and/or midsystolic notching, elevated early diastolic pulmonary regurgitation velocity, dilated inferior vena cava with reduced collapsibility, increased right atrial size): low probability of PH if peak TRV ≤ 2.8 m/s or not measurable and absence of indirect signs of PH, intermediate probability of PH if peak TRV ≤ 2.8 m/s or not measurable but indirect signs of PH or peak TRV 2.9–3.4 m/s but absence of indirect signs of PH, and high probability if peak TRV 2.9–3.4 m/s in combination with indirect signs of PH or peak TRV >3.4 m/s with or without indirect signs (11). This approach is accepted in the context of the “old” ESC/ERS 2015 PH definition as the Gold standard (PH: mPAP ≥25 mmHg). It is currently unknown whether a re-calibration is required when using the new PH definition (PH: mPAP >20 mmHg + additional criteria, see above).

There are algorithms composed of clinical parameters and non-invasive findings for the discrimination between pre- and post-capillary PH (59–61). Table 3 summarizes features favoring pre-capillary or post-capillary PH. A mildly reduced LVEF per se is no proof for post-capillary PH, and therefore attention must be given to markers of high left sided-filling pressures such as left ventricular diastolic dysfunction and LA dilatation/dysfunction. A high peak early mitral inflow velocity to peak early mitral annular velocity (E/e′) has turned out as a useful marker of a post-capillary pathology although studies on the correlation between E/e′ and left ventricular end-diastolic pressure or mPAWP in patients with preserved LVEF have revealed mixed results (62). Overall, the best predictors of pre-capillary PH include a small left LA (59, 61), a dilated RV (60, 61), a clearly visible D-shape of the left ventricle (60), a notch in the PW Doppler signal of the PA or a short acceleration time of <80 ms (59). The areas under the curve for these scores to predict pre-capillary PH range from 0.76 (60) to 0.93 (61). Still, only right heart catheterization can definitely make a diagnosis of PH and establish the underlying hemodynamic constellation (pre-capillary vs. post-capillary PH).

**Table 3:** Clinical features echo findings favoring pre-capillary or post-capillary pulmonary hypertension (PH).

	**Pre-capillary PH**	**Post-capillary PH**
**Clinical features**		
Atrial fibrillation^c^	No	Yes
Obesity/Diabetes^c^	No	Yes
Coronary artery disease	No	Yes
**Echocardiography**		
LV+LA area < RV+RA area^b^	Yes	No
Apex-forming RV^b^	Yes	No
RV end-diastolic area^c^	↑	↓
LV mass^c^	↓	↑
LV eccentricity index (degree of LV “D-shape”)^b^	↑	~1.0
E/e′^a,b^	↓	↑
LA area (apical for chamber view)^c^	↓	↑
LA anteroposterior diameter (parasternal long axis view)^a^	<3.2 cm	>4.2 cm
Mitral regurgitation	No/little	Little to severe
Peak TRV/VTI RVOT	↑	Normal/↓
Mid-systolic notch in pulmonary artery pulsed-wave Doppler signal or acceleration time <80 ms^a^	Yes	No
IVC diameter >20 mm without inspiratory collapse ( ≤ 50%)^b^	Yes	No

*E/e′, ratio of the peak early diastolic transmitral velocity to the peak early diastolic mitral annular velocity (ideally assessed at the lateral annulus); IVC, inferior vena cava; LA, left atrium; LV, left ventricle/ventricular; RA, right atrium/atrial; RV, right ventricle/ventricular; TRV, tricuspid regurgitation velocity; VTI RVOT, velocity time integral in the right ventricular outflow tract. ↑, large/high; ↓, small/low*.

a *Parameters included in the score by Opotowsky et al. (59)*.

5 *Parameters included in the score by D'Alto et al. (60)*.

c *Parameters included in the score by Berthelot et al. (61)*.

**Figure 2:**
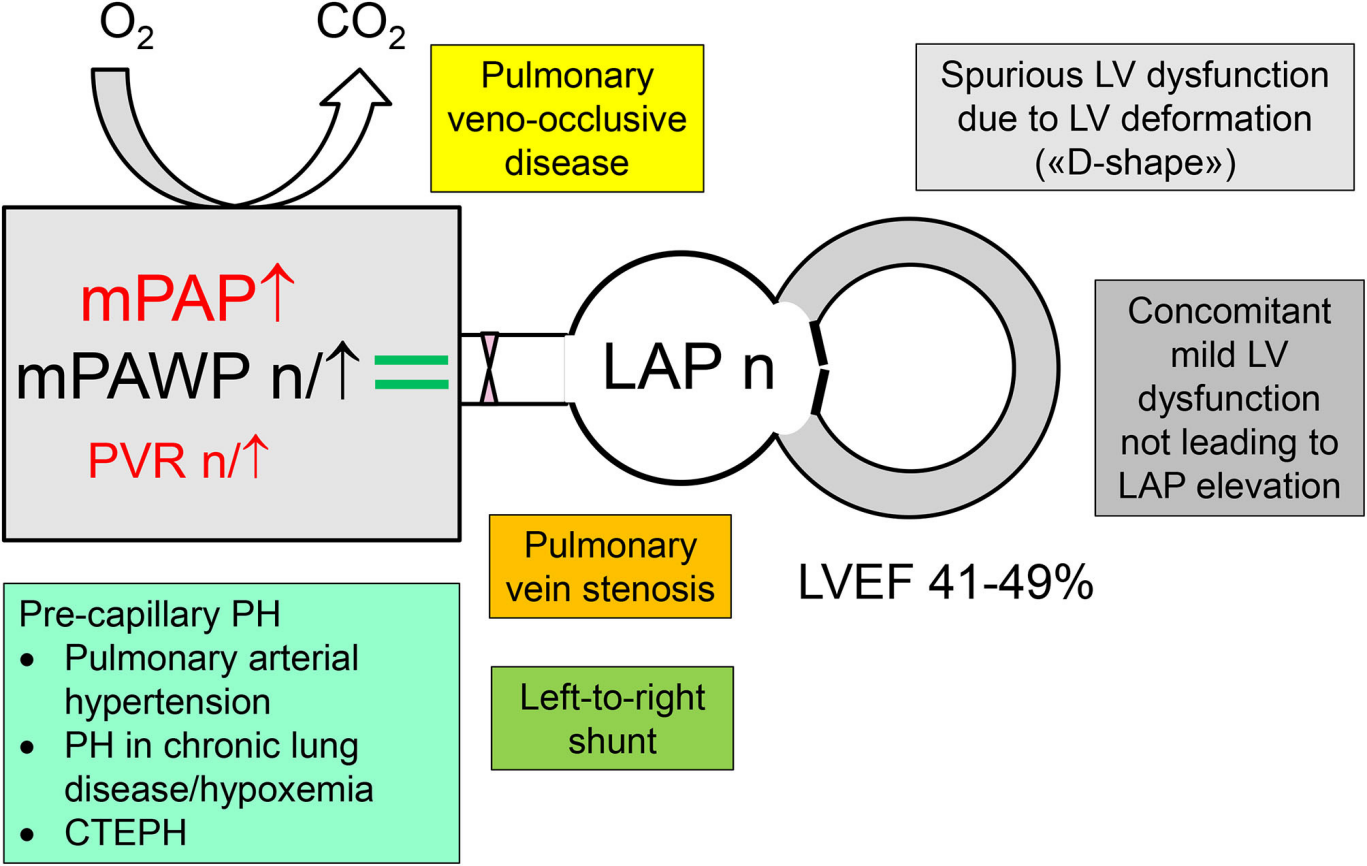
Differential diagnosis of pulmonary hypertension (PH) in patients with a left ventricular ejection (LVEF) in the mid-range of 41–49% but normal left atrial pressure (LAP), i.e., non-group 2 PH. CTEPH, chronic thromboembolic pulmonary hypertension; mPAP, mean pulmonary artery pressure; mPAWP, mean pulmonary artery wedge pressure; PVR, pulmonary vascular resistance.

In a patient with LVEF 41–49% and intermediate or high likelihood of PH, PH can be the consequence of LV dysfunction with LA pressure elevation, i.e., HFmrEF with group 2 PH, or this maybe a non-group 2 PH that co-exists with mild left ventricular dysfunction (Figures 1, 2). Measurement of natriuretic peptides will often not be helpful for discrimination, because elevated B-type natriuretic peptide (BNP) or N-terminal-proBNP (NT-proBNP) plasma concentrations can be the consequence of increased left ventricular wall stress (63) and thereby point toward left ventricular disease as the driver of symptoms (i.e., HFmrEF), but can also result from RV stress in case of pre-capillary PH (28).

**Figure 3:**
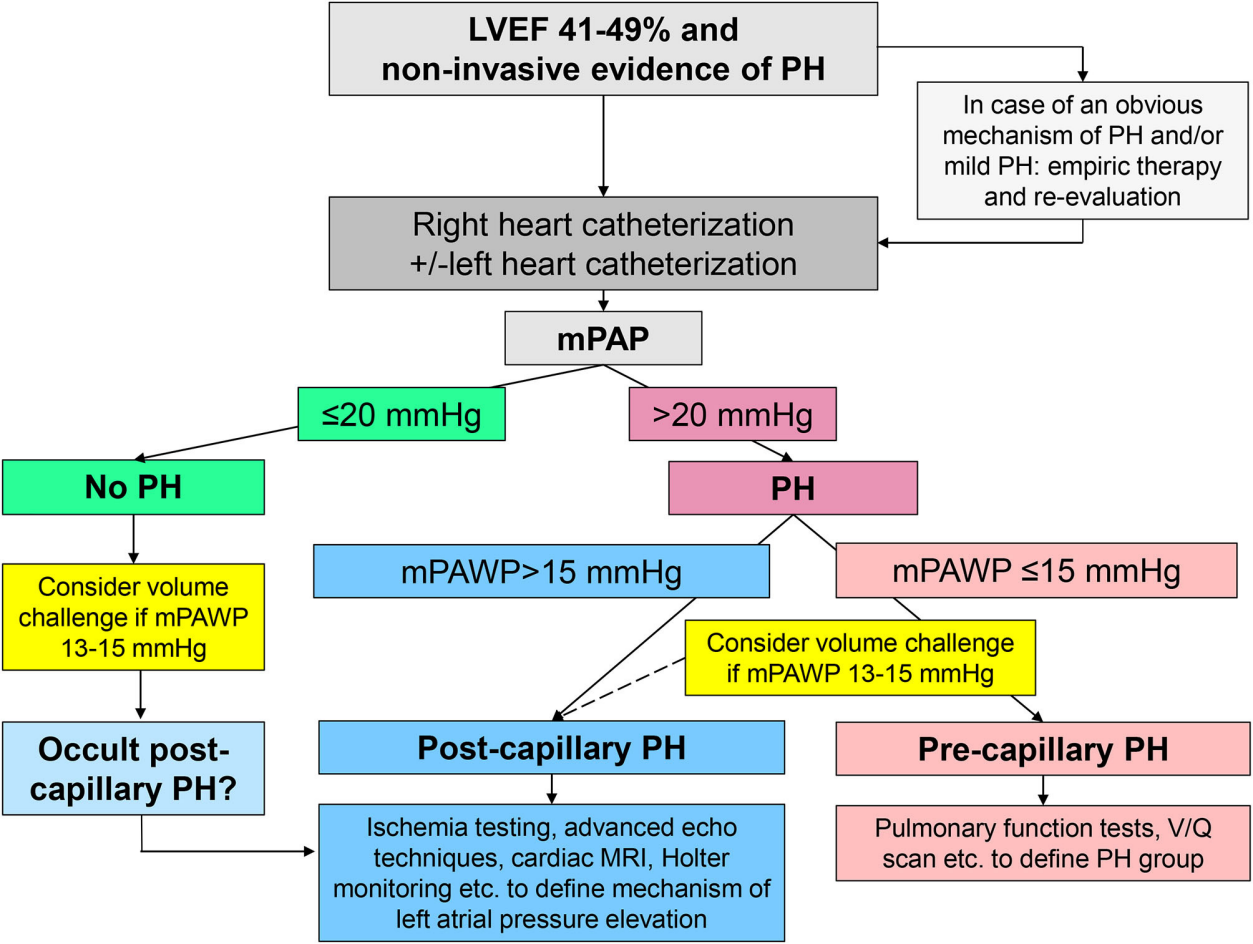
Schematic representation of the non-invasive and invasive work-up in patients with mid-range left ventricular ejection fraction (LVEF) of 41–49% and evidence of pulmonary hypertension (PH). mPAP, mean pulmonary artery pressure; mPAWP, mean pulmonary artery wedge pressure; MRI, magnetic resonance imaging; V/Q scan, ventilation perfusion scintigraphy.

**Figure 4:**
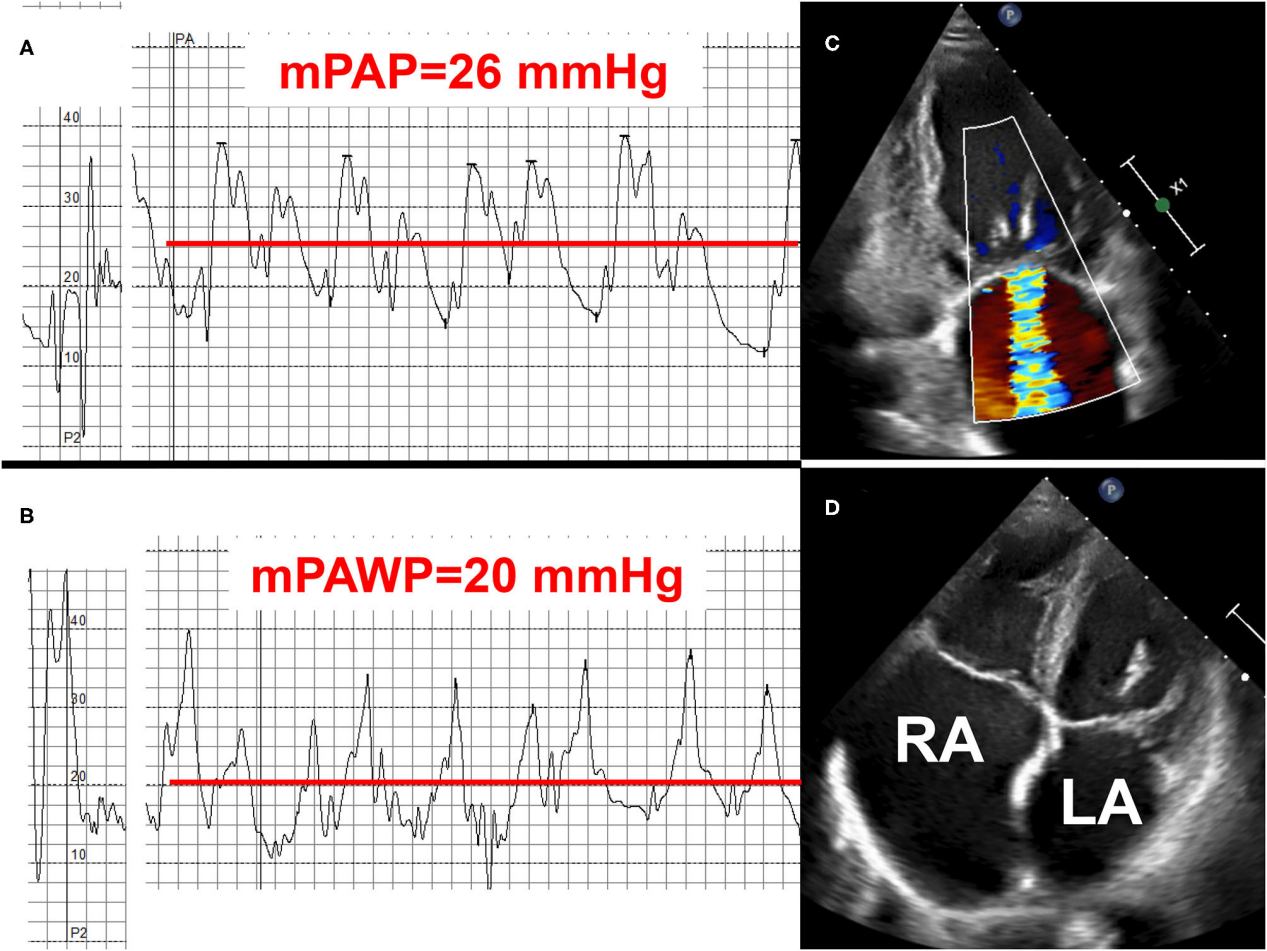
Example 1 of a patient with a mid-range left ventricular ejection fraction (LVEF) and pulmonary hypertension (PH): 78-year old female with permanent atrial fibrillation and coronary artery disease with previous myocardial infarction and percutaneous intervention of the occluded left circumflex artery (LCX). NYHA class II. LVEF 48%, moderate mitral regurgitation (MR), biatrial dilatation (LA: left atrium, RA: right atrium). Mean pulmonary artery pressure (mPAP): 26 mmHg **(A)**, mean pulmonary artery wedge pressure (mPAWP) 20 mmHg **(B)**, pulmonary vascular resistance: 1.7 Wood units. Left atrial pressure elevation and isolated post-capillary PH, respectively, are most likely multifactorial [left ventricular dysfunction, functional MR due to distorted left ventricular geometry after LCX infarct and atrial/annulus dilatation **(C)**, left atrial dysfunction in the context of atrial fibrillation **(D)**]. Management with loop diuretics, spironolactone, candesartan or sacubitril/valsartan, and betablocker. Rhythm control of atrial fibrillation may be considered but may not be successful; no evidence-based indication for mitral valve repair.

**Figure 5:**
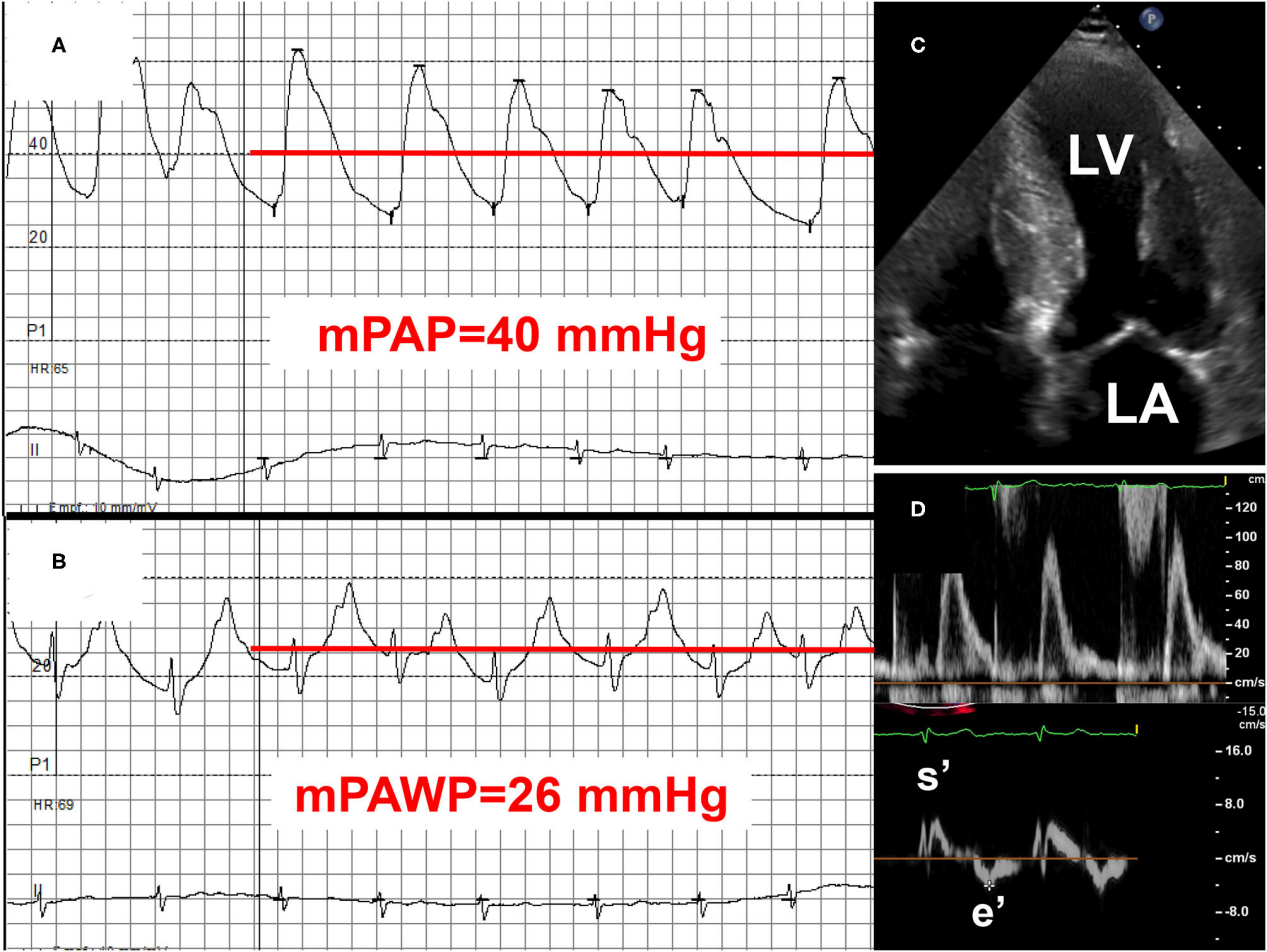
Example 2 of a patient with a mid-range left ventricular ejection fraction (LVEF) and pulmonary hypertension (PH): 75-year old man with transthyretin cardiac amyloidosis (positive technetium pyrophosphate scan). NYHA class III. LVEF 46%. Mean pulmonary artery pressure (mPAP): 40 mmHg **(A)**, mean pulmonary artery wedge pressure (mPAWP): 26 mmHg **(B)**, pulmonary vascular resistance: 2.5 Wood units. Left atrial pressure elevation is due to myocardial amyloid deposition **(C)** with significant systolic [long axis function, reduced systolic mitral annular velocity (s′)] and diastolic dysfunction [markedly reduced peak early diastolic mitral annular velocity (e′); **(D)**]. Management primarily with loop diuretics and spironolactone; tafamidis may be considered but may have limited effect in this advanced disease stage (NYHA III). LA, left atrium; LV, left ventricle.

**Figure 6:**
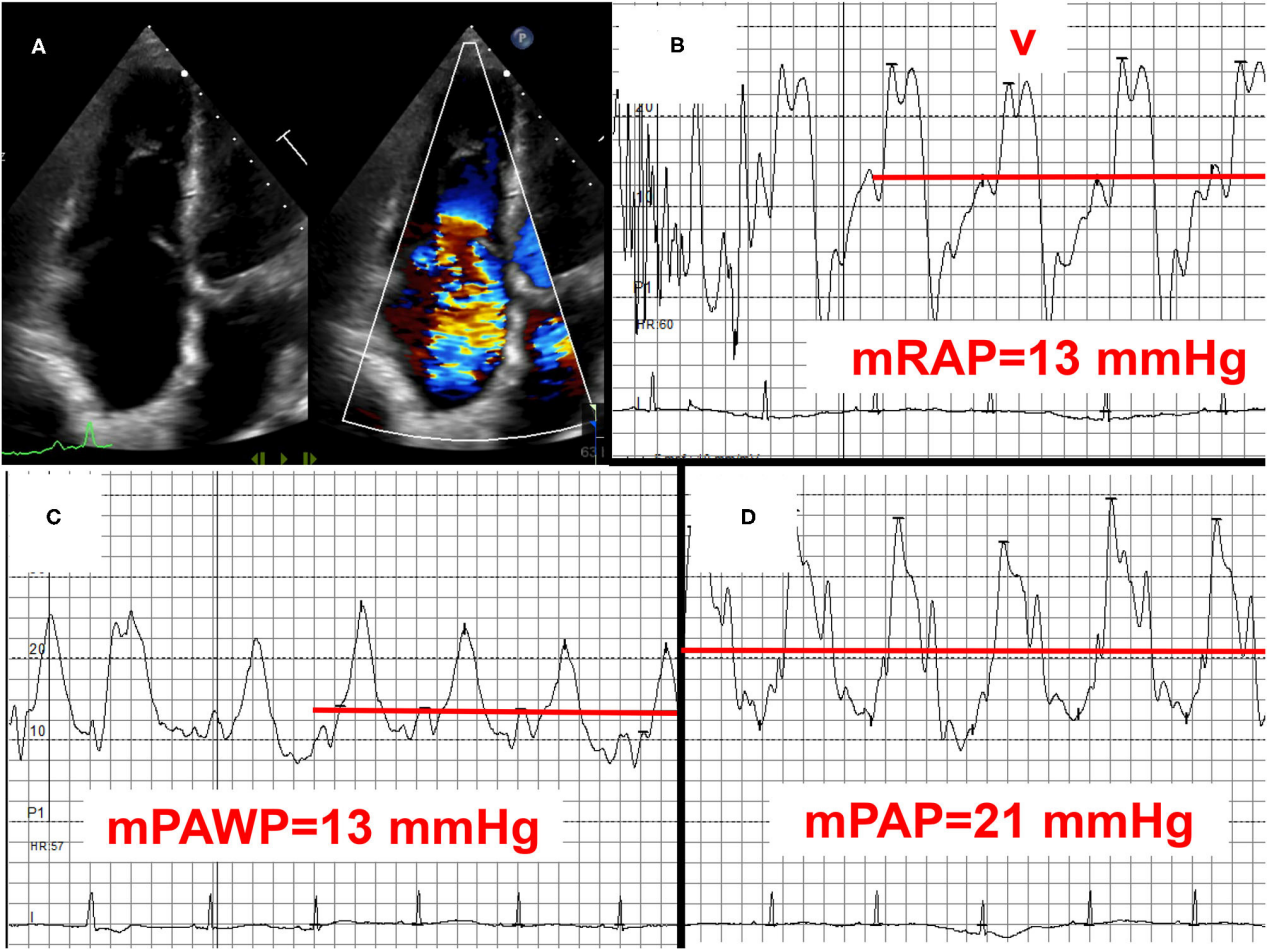
Example 3 of a patient with a mid-range left ventricular ejection fraction (LVEF) and pulmonary hypertension (PH): 83-year old female with permanent atrial fibrillation, previous aortic valve replacement, and coronary artery disease. LVEF 45%, normally functioning aortic bioprothesis, mild to moderate mitral regurgitation, and severe tricuspid regurgitation **(A)** with signs of right heart failure and high right atrial pressure with high V waves **(B)**. Mean pulmonary artery wegde pressure (mPAWP): 13 mmHg **(C)**, mean pulmonary artery pressure (mPAP): 21 mmHg **(D)**. After coronary angiography (50 ml of contrast) rise in mPAWP to 18 mmHg and mPAP to 26 mmHg. The patient has occult post-capillary PH (2016 ESC/ERS definition)/mild post-capillary PH (2018 definition) in the context of left ventricular systolic and diastolic dysfunction. The relatively mild extent of PH does not fully explain right ventricular dilation and severe tricuspid regurgitation. Severe tricuspid regurgitation is most likely the effect of atrial fibrillation predominantly affecting the tricuspid annulus. Management with loop diuretics and spironolactone. The role of transcatheter tricuspid valve repair/replacement has not been defined yet. mRAP, mean right atrial pressure.

The classical class I indication for right heart catheterization in patients with group 2 PH is in the context of transplant evaluation (11). Guidelines state that right heart catheterization may also be considered (class IIb indication) in patients with left heart diseases and suspected PH to assist in the differential diagnosis and support treatment decisions (11). If non-invasive imaging clearly points to group 2 PH, treatment can be established, in particular euvolemia must be achieved. Depending on the context and the extent of the suspected PH, right heart catheterization may still be performed early in the diagnostic pathway to clarify the hemodynamic constellation, and additional tests will be performed depending on the result (pre- vs. post-capillary PH) (Figure 3). In patients with a borderline hemodynamic constellation (i.e., mPAWP 13–15 mmHg), there may be occult post-capillary PH following prolonged fasting or diuretic therapy, and a volume or exercise challenge may be required to unmask group 2 PH (14). In patients with post-capillary PH, the key mechanism of LA pressure and mPAWP elevation, respectively, has to be identified as a basis for appropriate therapy (Table 2). In Figures 4–6, three examples of patients with HFmrEF and PH are presented. These very different cases highlight the heterogeneity within the HFmrEF population and the challenges associated with diagnosis and therapy.

Notably, most patients with an LVEF between 41 and 49% (typically after myocardial infarction) seen in daily practice are not symptomatic from HF, and therefore mechanisms for dyspnea other than the mildly reduced LVEF have to be carefully looked for (Table 4). Patients with pre-capillary PH and LVEF 41–49% need a work-up to define the underlying PH group (pulmonary arterial hypertension, PH in context of lung disease/chronic hypoxia, chronic thromboembolic PH) as this has may have direct therapeutic consequences. The mildly reduced LVEF in these cases typically results from LV deformation due to RV pressure overload (“D-shape”) or represents a concomitant mild LV disease, which however is not hemodynamically predominant.

**Table 4:** Differential diagnosis of pulmonary hypertension (PH) and mid-range/“mildly reduced” left ventricular ejection fraction (LVEF).

	**Characteristics**	**Hemodynamics**	**Diagnostic approach**
HFmrEF with group 2 PH (cf. Table 1)	PH as consequence of HFmrEF	2015: mPAP ≥25 mmHg and mPAWP >15 mmHg 2018: mPAP >20 mmHg and mPAWP >15 mmHg	Identification of treatable mechanisms of HF: ischemia, atrial fibrillation, primary valve disease, systemic disease; RHC if hemodynamic constellation unclear
Group 1 PH and LVEF 41–49%	Pulmonary arterial hypertension with concomitant unrelated mild LV disease (or “only” LV deformation due to flattening of the interventricular septum)	2015: mPAP ≥25 mmHg and mPAWP ≤ 15 mmHg 2018: mPAP >20 mmHg, mPAWP ≤ 15 mmHg, and PVR ≥3 WU	RHC, ventilation/perfusion scintigraphy, lung function, sleep study, evaluation of specific etiologies (liver disease, connective tissue disease, etc.)
Group 3 PH and LVEF 41–49%	PH in the context of chronic lung disease/chronic hypoxemia combined with mild LV dysfunction (e.g., previous myocardial infarction)	2015: mPAP ≥25 mmHg and mPAWP ≤ 15 mmHg 2018: mPAP >20 mmHg, mPAWP ≤ 15 mmHg, and PVR ≥3 WU	Lung function tests including CO diffusion, CT scan, sleep study. RHC only in selected cases Identification of the concomitant cardiac disease, e.g., cardiac MRI and coronary angiography in case of suspected coronary artery disease
Group 4 PH and LVEF 41–49%	Chronic thromboembolic PH combined with mild LV disease (or “only” LV deformation due to flattening of the interventricular septum)	2015: mPAP ≥25 mmHg and mPAWP ≤ 15 mmHg 2018: mPAP >20 mmHg, mPAWP ≤ 15 mmHg, and PVR ≥3 WU	RHC, ventilation/perfusion scintigraphy, pulmonary angiography
Left-to-right shunt with/without mild LV disease	Atrial septal defect or abnormal pulmonary venous drainage	2015: mPAP ≥25 mmHg, pulmonary blood flow↑↑ 2018: mPAP >20 mmHg, pulmonary blood flow ↑↑	TTE and TEE and CT scan to identify the shunt, RHC
High output HF	Liver disease, severe anemia, or other high-output condition associated with mild LV dysfunction	2015: mPAP ≥25 mmHg, mPAWP >15 mmHg, and cardiac index↑↑ 2018: mPAP >20 mmHg, mPAWP >15 mmHg, PVR <3 WU, and cardiac index↑↑	Internistic work-up, TTE, RHC

*LV, left ventricular; mPAP, mean pulmonary artery pressure; mPAWP, mean pulmonary artery wedge pressure; PVR, pulmonary vascular resistance; RHC, right heart catheterization; TEE, transesophageal echocardiography; TTE, transthoracic echocardiography. ↑↑, substantially increased*.

## Treatment of PH in HFmrEF

The general principle applying to the treatment of PH in HF is to treat the underlying cardiac disease and its risk factors, particularly the metabolic syndrome, and to identify and treat co-morbidities that may also lead to PH such as chronic obstructive lung disease and obstructive sleep apnea (1, 2, 11). For patients with HFrEF, treatment is well-defined and includes several drugs with different mechanisms of action with established effect on symptoms and prognosis (7). Wireless pulmonary artery pressure monitoring data have shown that PA pressure can be effectively lowered by guideline-directed disease-modifying therapy and diuretics (64). In contrast, there is still no treatment, which has been shown to improve prognosis in patients with HFpEF (7). Diuretics are recommended for the management of congestive symptoms in these patients (7). However, given the typically small left ventricular volumes (concentric remodeling) and the steep end-diastolic pressure-volume relationship there is a relatively narrow therapeutic window for the use of diuretics. Diuretics will efficiently reduce LVEDP, LAP, mPAWP, and mPAP but these patients are also at risk for overtreatment with hypotension and renal failure (1). In patients with “true” HFpEF (i.e., LVEF ≥50%), studies testing drugs with proven survival benefit in HFrEF (inhibitors of the renin-angiotensin system, spironolactone) have failed to show any benefit (1). For HFmrEF patients, no specifically designed trials have been performed (6), and treatment of these patients is currently not well-defined. However, subgroup and *post-hoc* analyses of three large “HFpEF studies” using variable LVEF cut-offs for inclusion and evaluating the effect of candesartan vs. placebo (LVEF >40%) (65), spironolactone vs. placebo (LVEF ≥45%) (66), and sacubitril/valsartan vs. valsartan alone (LVEF ≥45%) (27) have revealed that patients fitting into the current HFmrEF range (i.e., LVEF 40–49 or 45–49%) may benefit from these three drugs. In addition, a recent meta-analysis found evidence of a benefit of betablocker therapy in HFmrEF patients (67). Thus, we suggest that patients with HFmrEF and post-capillary PH should be treated with these drugs and loop diuretics as needed. The recommendations of the 2021 ESC HF guidelines on HFmrEF are not published yet and may be more reluctant. Still, we think that the use of these potentially effective drugs should be considered if there PH, i.e., a manifestation of advanced HF. In addition, specific mechanisms of LA pressure and mPAWP elevation must be targeted, e.g., tachycardia, atrial fibrillation, myocardial ischemia, infiltrative diseases, and functional MR. Several mechanism may contribute to LA pressure elevation (Figure 1), and careful non-invasive and invasive diagnostic evaluation is prerequisite for a tailored therapy (Table 2) (27, 65–71). The role of AF seems to be particularly important. Atrial fibrillation seems to be a key factor in the pathophysiology of PH in HFpEF and HFmrEF as discussed above and is associated with the combined endpoint of all-cause mortality and heart failure hospitalizations in HFpEF and HFmrEF but not in HFrEF (72). A recent study has shown a favorable effect of successful catheter ablation on exercise hemodynamics (reduction in peak exercise mPAWP) and quality of life in patients with HFpEF (71). It is speculated that rhythm control of AF may be a very important strategy to treat or prevent PH in patients with HFpEF und HFmrEF.

In contrast to patients with HFrEF, evidence for the utility of device therapy for the treatment of HFmrEF and HFpEF is scarce. This refers to defibrillators (except for secondary prevention), cardiac resynchronization, and transcatheter mitral valve repair. In transplant candidates with advanced HFrEF (mean LVEF 18%) and CpcPH without acute reversibility, left ventricular unloading by implantation of an assist device has been shown to result in a reduction in PVR from 5.1 to 2.0 WU within 6 weeks (73). It is unknown how the pulmonary vasculature is remodeling in this context, and whether this approach would also be successful in HFpEF and HFmrEF. However, the latter two groups are rarely candidates for transplantation. As an important exception regarding the applicability of devices, the concept of an intraatrial shunt device for LA decompression has been successfully tested in HFpEF and HFmrEF (74, 75). In patients with LVEF ≥40%, this device led to a similar reduction in exercise mPAWP (driven by the pre-implant exercise mPAWP to right atrial pressure gradient) in patients with HFpEF and HFrEF with mPAWP >15 mmHg at rest or >25 mmHg on exercise (76). The mean resting mPAWP and mPAP in the study population were 17 and 24 mmHg indicating that the population included a relevant number of patients with post-capillary PH (74, 76). Interestingly, a *post-hoc* analysis of hemodynamics in 79 patients treated with the intraatrial shunt device (mean LVEF 47%; 68% of patients with LVEF 40–49%, mean mPAP and mPAWP 26 and 18 mmHg, respectively) revealed that the 27% increase in pulmonary flow at rest was accompanied by a 17% reduction in PVR and a 24% increase in pulmonary artery compliance (77). Similar changes were observed during exercise. It was speculated that the increase in pulmonary flow and oxygen content may have led to beneficial effects on the pulmonary vasculature. Thus, this therapeutic approach may be relevant for the management of patients with PH in the context of HFmrEF. Importantly, the mean PVR was 1.5 WU, and patients with a PVR 4 ≥WU were excluded (77).

Patients with HFmrEF and PH (i.e., post-capillary PH) may benefit from a particularly aggressive use and combination of the available treatments including diuretics. There are, however, no established drugs specifically targeting PH in HF in general and also in HFmrEF. Only a few studies have studied the effect of specific pulmonary arterial hypertension-targeted therapeutics in patients with HFpEF or HFmrEF and PH (Table 5) (78–87). In general, the use of pulmonary vasodilatators did not improve hemodynamics or exercise capacity. The most promising substance in this context is the phosphodiesterase inhibitor sildenafil. In a study among patients with HFpEF or HFmrEF (LVEF ≥45%) and IpcPH or mild CpcPH (mPAWP = 20 mmHg, PVR = 2.6 WU; 35% with PVR >3 WU) sildenafil compared to placebo exerted no effect on mPAWP, cardiac output and functional capacity (81). However, Guazzi et al. (79) reported a substantial reduction in mPAWP and PVR as well as an improvement in TAPSE in HFpEF patients (LVEF cut-off for inclusion: ≥ 50%) with somewhat higher PVR (around 3.6 WU) and poor RV function (TAPSE of 11 mm). A second study in patients with HFpEF and CpcPH found a benefit of sildenafil vs. placebo in terms of NYHA class, 6-min walking distance, and sPAP (82). However, this was a non-invasive study, and both the hemodynamic inclusion criteria and the endpoint (sPAP) were assessed by echocardiography. At the moment, it remains unclear whether patients with HFmrEF (and HFpEF) and more severe CpcPH (i.e., higher PVR) and RV dysfunction may benefit from specific pulmonary arterial hypertension-targeted therapeutics, in particular phosphodiesterase inhibitors. The PASSION trial evaluating the impact of tadalafil on clinical endpoints in patients with HFpEF (LVEF ≥50%) and CpcPH (mPAP ≥25 mmHg, mPAWP >15 mmHg, PVR >3 WU) is ongoing and will provide relevant information with potential implications for HFmrEF patients (50).

**Table 5:** Studies on pulmonary arterial hypertension targeted therapeutics in patients with heart failure (HF) with preserved (HFpEF) or mid-range (HFmrEF) left ventricular ejection fraction (LVEF) with or at risk for pulmonary hypertension.

	**Population**	**Intervention**	**Main results**
Andersen et al. (78)	Inclusion criteria: Recent myocardial infarction, revascularized, LVEF ≤ 45%, E/e′≥8, LAVI ≥34 ml/m^2^, Hemodynamics: mPAP ≈ 20 mmHg, mPAWP ≈ 13 mmHg (*n* = 70)	Sildenafil 3 ×40 mg vs. placebo for 9 weeks	Trend toward exercise mPAWP reduction CO↑ and SVR ↓
Guazzi et al. (79)	Inclusion criteria: LVEF ≥50% sPAP>40 mmHg (*n* = 44) Hemodynamics: mPAP ≈ 37 mmHg, mPAWP ≈ 22 mmHg, PVR ≈ 3.5 WU	Sildenafil 3 ×50 mg vs. placebo for 6 months	mPAP↓ mPAWP↓ Cardiac index↑ Right ventricular function↑
Redfield et al. (80)	Inclusion criteria: LVEF ≥50%+elevated NT-proBNP or non-invasive evidence of elevated filling pressures (*n* = 216) Hemodynamics: not measured	Sildenafil 3 ×20 mg for 12 weeks, then 3 ×60 mg vs. placebo for 12 weeks	No effect on peak VO_2_ and 6-min walking distance
Hoendermis et al. (81)	Inclusion criteria: LVEF ≥45%, mPAP >25 mmHg, mPAWP >15 mmHg Hemodynamics: mPAP ≈ 35 mmHg mPAWP ≈ 20 mmHg	Sildenafil 3 ×60 mg vs. placebo for 12 weeks	No effect on mPAP, mPAWP, CO, and peak VO_2_
Belyavskiy (82)	Inclusion criteria: LVEF >50%, sPAP >40 mmHg, PVR >3 WU and/or transpulmonary gradient >15 mmHg (all assessed by echocardiography) Hemodynamics: not measured	Sildenafil 3 ×25 mg for 3 months, followed by 3 ×50 mg for 3 months vs. placebo	Improvement in NYHA class and 6 min walking distance, reduction in sPAP
Bonderman et al. (83)	Inclusion criteria: LVEF>50%, mPAP ≥25 mmHg, mPAWP >15 mmHg (*n* = 39) Hemodynamics: mPAP ≈ 35 mmHg mPAWP ≈ 20 mmHg	Single dose of Riociguat of 0.5 mg, 1.0 mg, or 2.0 mg vs. placebo	No effect on mPAP after 6 h Stroke volume↑ Systolic blood pressure↓ Right ventricular end-diastolic area
Bermejo et al. (84)	Inclusion criteria: PH post valve surgery but no significant valvular dysfunction, mPAP >30 mmHg Hemodynamics: mPAP ≈ 38 mmHg mPAWP = 23 mmHg PVR ≈ 3.3 WU (*n* = 200)	Sildenafil 3 ×40 mg (3 ×20 mg for selected patients) vs. placebo for 6 months	Worse composite clinical score (death, hospitalization for HF, change in functional class, patient global self assessment) in the sildenafil treated patients
Zile et al. (85)	Inclusion criteria: LVEF ≥50% + evidence of concentric remodeling and/or LV diastolic dysfunction E/e′ 14, peak TRV 2.7 m/s (*n* = 192) Hemodynamics: not measured	Sitaxsentan 100 mg/d vs. placebo for 24 weeks (2:1 randomization)	Increase in treadmill time, no effect on quality of life, death, HF hospitalization
Koller et al. (86)	Inclusion criteria: HFpEF (ESC 2016 definition) and mPAP ≥25 mmHg, mPAWP >15 mmHg Hemodynamics: mPAP ≈ 38 mmHg mPAWP ≈ 21 mmHg PVR ≈ 4.2 WU (*n* = 20)	Bosentan 2 ×62.5 mg for 4 weeks, 2 ×125 mg for 8 weeks vs. placebo	Higher pulmonary artery and right atrial pressure (echo) and worsening 6 min walking distance in Bosentan group
Vachiery et al. (87)	Inclusion criteria: Combined pre-capillary and post-capillary PH (mPAP ≥25 mmHg, mPAWP >15 mmHg but <25 mmHg, DPG ≥7 mmHg and PVR >3 WU), LVEF ≥30% (≥50%: 81%, <50%: 19%) Hemodynamics mPAP ≈ 47 mmHg, mPAWP ≈ 20 mmHg, PVR ≈ 5.8 WU) (*n* = 63)	Macitentan 10 mg vs. placebo for 12 weeks	Trend toward more fluid retention in the Macitenan group No effect on mPAWP and PVR

*DPG, diastolic pressure gradient; ESC, European Society of Cardiology; LAVI, left atrial volume index; LV, left ventricular; mPAP, mean pulmonary artery pressure; mPAWP, mean pulmonary artery wedge pressure; NT-proBNP, N-terminal-pro-B-type natriuretic peptide; NYHA, New York Heart Association; peak VO_2_, peak oxygen consumption; PVR, pulmonary vascular resistance; sPAP, systolic pulmonary artery pressure; E/e′, ratio of peak early diastolic transmitral velocity to peak early diastolic mitral annular velocity; TRV, tricuspid regurgitant velocity*.

## Future perspectives

Intense research will be required to define the key mechanism underlying the pathophysiology of (a) HFmrEF and (b) PH in HFmrEF. This will lead to a refinement of the definition, the diagnostic criteria and the therapeutic approach. Very recently, a universal definition and classification of HF has been proposed (10). In this position paper issued by all of the important HF societies, a fourth HF class has been suggested: HF with improved LVEF, i.e., HF with an initial LVEF ≤ 40% and an improvement by at least 10 percent points to an LVEF >40% (10). Whether or not this group of patients requires a different treatment than patients with (stable) HFmrEF or HFpEF will have to be shown. The 2021 ESC HF guidelines are about to be published and will define the diagnostic criteria and thereby probably follow the universal definition and classification of HF (10). For the treatment of HFmrEF in general and most likely also PH in HFmrEF the data on the effect of sodium-glucose co-transporter 2 (SGLT2) inhibitors will be very important (88, 89). Mechanistic studies suggest that SGLT2 inhibitors exhibit favorable effects on cardiac inflammation and fibrosis and thereby cardiac remodeling also in subjects with preserved LVEF (90). Importantly, significant hemodynamic effects, i.e., reduction in mPAWP (91) and PA pressures (92) have been demonstrated for SGLT2 inhibitors, most likely indirectly via beneficial effects on cardiac structure and function but also directly via the diuretic properties (93) of these drugs. The baseline characteristics of the Empagliflozin Outcome Trial in Patients With Chronic Heart Failure With Preserved Ejection Fraction (EMPEROR-Preserved) have already been published (89): the LVEF cut-off for study inclusion was ≥40%, and the mean baseline LVEF is 54 ± 9% indicating that the trial also included a relevant number of HFmrEF patients (89) and that the results of this trial will be highly relevant for the setting of HFmrEF in general and also HFmrEF with PH. The three cases presented in Figures 4–6 highlight however, that management of these patients is challenging, that a clear guideline-based recommendation will not available for all scenarios, and that treatment must always be tailored based on a careful non-invasive and often also invasive assessment.

Apart from the treatment of the underlying left heart pathology (i.e., HFmrEF) there is currently intense research investigating novel treatments targeting the pulmonary vasculature directly (5, 94). Approaches currently under study for patients with PH in the context of HF include among others the β3 adrenergic receptor agonist mirabegron, the antifibrotic agent PBI 40–50, the rho kinase inhibitor fasudil, the calcium sensitizer levosimendan, oral sodium nitrite, and catheter-based pulmonary artery denervation (5, 94). It is likely that only certain pulmonary vascular phenotypes with PH in HFmrEF or HFpEF will derive benefit from such an approach. Only studies with detailed clinical, biochemical, and hemodynamic phenotyping will be able to define whether there is a subset of patients with PH in the context of HFmrEF who will benefit from specific pulmonary arterial hypertension-targeted therapeutics.

## Conclusions

Heart failure with mid-range LVEF in general, and PH in HFmrEF in particular, are entities that have been incompletely characterized. Cross-sectional studies suggest that HFmrEF patients are overall characterized by a left heart phenotype which is intermediate between HFrEF and HFpEF. With regards to the pathophysiology of PH the available data suggest that there are many similarities with HFpEF. In clinical practice, patients with shortness of breath, an LVEF in the mid-range of 41–49% and evidence of PH represent a diagnostic challenge. First, a careful differentiation between post- and pre-capillary PH is required. Second, in patients with post-capillary PH the predominant mechanism of LA pressure elevation has to be identified as this will represent the primary target for therapy. In terms of medical therapy, there is some evidence for a benefit of classical HFrEF therapeutics, i.e., angiotensin receptor blockers, spironolactone, sacubitril/valsartan, and betablockers for HFmrEF and presumably also for HFmrEF with PH. However, at the moment, this is still speculative, and substantial additional research will be required to define the optimal management of these patients.

## Author Contributions

MM: conception, collection of data, writing of first draft, and finalization of paper. LW, MB, RB, LJ, and HR: critical revision and final approval. All authors contributed to the article and approved the submitted version.

## Conflict of Interest

The authors declare that the research was conducted in the absence of any commercial or financial relationships that could be construed as a potential conflict of interest.
